# Vaccine hesitancy among parents of children with chronic diseases of different pathophysiology: a cross-sectional study in Sivas, Türkiye

**DOI:** 10.1186/s12889-025-22797-y

**Published:** 2025-05-07

**Authors:** Ayça Kömürlüoğlu, Nurullah Çelik, Ayla Uzun Çiçek, Siddika Songül Yalçın

**Affiliations:** 1 Institude of Child Health, Department of Social Pediatrics, Hacettepe University, Ankara, Türkiye; 2 Faculty of Medicine, Department of Pediatrics, Sivas Cumhuriyet University, Sivas, Türkiye; 3 Faculty of Medicine, Department of Pediatric Endocrinology, Sivas Cumhuriyet University, Sivas, Türkiye; 4 Faculty of Medicine, Department of Child and Adolescent Psychiatry, Sivas Cumhuriyet University, Sivas, Türkiye

**Keywords:** Vaccine hesitancy, Vaccination, T1DM, Vaccine refusal, ASD, ADHD

## Abstract

**Background:**

Vaccine hesitancy (VH) and vaccine refusal are increasing globally, posing a significant challenge to public health. This study aimed to evaluate VH and associated factors in parents of children with different chronic conditions, comparing them to a control group of healthy children.

**Methods:**

This cross-sectional study included mothers of children aged 6 to 12 years, diagnosed with Autism Spectrum Disorder (ASD), attention deficit hyperactivity disorder (ADHD), type 1 diabetes mellitus (T1DM), congenital heart disease (CHD), congenital hypothyroidism (CH) and Familial Mediterranean Fever (FMF) and healthy children without chronic diseases. The study collected sociodemographic data, and parents completed the Parent Attitudes about Childhood Vaccines (PACV) survey and the Parental Attitude Research Instrument (PARI). Vaccine hesitancy was defined as a PACV score ≥ 50.

**Results:**

A total of 1163 participants were included, consisting of 546 children with chronic conditions. The overall VH rate was %14.7. Compared to control group, parents of children with T1DM had 3.3 times higher odds of VH, and parents of children with ASD had 1.8 times higher odds of VH. However, parents of children with CHD had lower odds of VH [OR: 0.38 (95% CI: 0.15–0.97)]. The most common reasons for VH were concerns about vaccine ingredients (40.2%) and fear of adverse events (22.5%). The primary suggested solution was receiving more information from healthcare professionals (33.3%). Factors such as having a child with a chronic condition, personal experience with vaccine adverse events, and reliance on the internet for vaccine information were associated with increased VH, whereas obtaining information from healthcare professionals was linked to lower VH. Higher parental democratic attitudes were associated with lower VH, while increased marital conflict was linked to higher VH.

**Conclusion:**

Addressing both informational gaps and psychosocial factors, such as marital conflict and democratic parenting attitudes, can enhance vaccine acceptance. Healthcare professionals should provide personalized guidance and resources to empower parents, enabling them to make informed vaccination decisions for high-risk groups such as children with chronic conditions.

**Supplementary Information:**

The online version contains supplementary material available at 10.1186/s12889-025-22797-y.

## Introduction

Vaccination is a safe, cost-effective public health achievement that saves millions of children’s lives annually by preventing vaccine-preventable diseases (VPDs) and reducing associated morbidity and mortality. Vaccination provides both individual and collective immunity, necessitating high vaccination rates with the vaccination schedule is of critical importance [1, 2]. However, vaccine hesitancy (VH) and vaccine refusal (VR) have been increasing globally, threatening herd immunity [3]. World Health Organization (WHO) describes VH “*a motivational state of being conflicted about*,* or opposed to*,* getting vaccinated; this includes intentions and willingness*” and VR as “*the act of not vaccinating children due to a decision to decline all vaccines”.* Some parents fully support and advocate for all vaccines, while others firmly refuse them [4]. A multitude of factors influence VH and parents vaccination decision, including social media, vaccine lobbies, influential leaders, religious, cultural, geographical, social, political, and economic factors, perceptions about the pharmaceutical industry, concerns about vaccine adverse events, lack of trust in vaccination [3–7]. The reasons identified in one-on-one interviews with hesitant parents are particularly valuable [7, 8].

A significant factor contributing to parents’ hesitancy regarding vaccines is the ongoing discourse surrounding the potential association between vaccines and autism. In 1998, Wakefield, a gastroenterologist, published a case series in The Lancet, asserting a link between the Measles-Mumps-Rubella (MMR) vaccine and autism [3]. Despite repeated assurances from health professionals and extensive epidemiological studies definitively disproving such a link, the paper caused widespread fear around the world. Vaccination rates have fallen and vaccination campaigns have been disrupted. After a decade of debate and investigation, Wakefield was found guilty of ethical, medical and scientific misconduct for conducting the study and publishing false data [3, 9]. However, since then, the claim of a relationship between vaccines and autism has been one of the leading reasons for VH. In fact, no scientific study has ever found a relationship between vaccines and autism [10, 11]. Evaluating vaccine acceptance of parents with children diagnosed with autism spectrum disorder (ASD) and examining the underlying reasons for VH are important research topics. Evidence indicates that younger siblings of children with ASD have lower vaccination rates, largely due to parental concerns based on the misconception that vaccination may have contributed to the older child’s diagnosis [12, 13].

In Türkiye, vaccination is voluntary, with the Expanded Immunization Program providing free vaccines against multiple infectious diseases including tuberculosis, diphtheria, pertussis, tetanus, poliomyelitis, measles, rubella, mumps, chickenpox, hepatitis A, pneumococcus, and Haemophilus influenzae type b. However, rising VH and VR are particularly concerning for children with chronic illnesses, who are at higher risk for VPDs. Zero-dose children in Türkiye has dropped from 3.2 to 0.9% over the last three decades [14]. However, VH and VR cases are increasing in our country at last years [15]. A drop in immunization rates below 95% can lead to outbreaks of VPDs, particularly measles, increased morbidity and mortality [16]. Children with chronic diseases are an important risk group for VPDs, especially those with respiratory, cardiovascular, liver, renal, and neoplastic diseases, and their vaccination on time and in accordance with the vaccination schedule has critical importance [17].

The first and important step in developing effective strategies for VH and VR is to understand the reasons and contexts that lead to vaccine acceptance, hesitancy, and refusal [18]. Parents of children with different health indicators may have different attitudes and behaviors about vaccination because they receive different stimuli about vaccination [17]. In this study, we aimed to determine vaccine acceptance in parents of children with diseases of different pathophysiology, to examine the differences according to the health status of children, and to evaluate the relationship with parental attitudes and VH. We hypothesize that parental vaccine acceptance for children with chronic diseases is influenced by the child’s health status, with parents of children with more severe or complex health conditions exhibiting higher levels of VH.

The key research questions we sought to address were: “How does the health status of the child influence parental vaccine acceptance, hesitancy, and refusal?”, “Are there significant differences in VH between parents of children with chronic diseases compared to those with acute or no health conditions?”, “What role do parental attitudes toward vaccination play in shaping vaccine acceptance or hesitancy, particularly for children with chronic diseases?”

By addressing these questions, the results of the present study contributes to the existing literature by examining the factors influencing vaccine acceptance, particularly in children with chronic diseases. By identifying these factors, the study will inform the development of tailored strategies to increase immunization rates in this high-risk group. Ultimately, this research seeks to enhance efforts to reduce VH and VR, strengthening public health initiatives and improving vaccine coverage in society.

## Materials and methods

### Study population and sampling

The study was conducted between 01.09.2021 and 01.09.2022. Patients between the ages of 6–12, years who admitted to Sivas Cumhuriyet University General Pediatrics, Pediatric Endocrinology, and Child and Adolescent Mental Health and Diseases Outpatient Clinics, diagnosed with ASD, attention deficit hyperactivity disorder (ADHD), type 1 diabetes mellitus (T1DM), congenital heart disease (CHD), congenital hypothyroidism (CH), and Familial Mediterranean Fever (FMF), were included in the study. Study groups are given in Table 1. The control group consisted of healthy children and their parents, who were matched with the study groups based on age, gender, and socio-demographic characteristics. Healthy controls were defined as children without any diagnosed chronic medical conditions.

**Table 1:** Classification of clinical conditions in the study population: grouping of study participants based on clinical and vaccination considerations

Grouping Based on Clinical and Vaccination Considerations	Included Clinical Conditions	Description
Chronic Condition with Potential Vaccine Hesitancy Group	Autism Spectrum Disorder (ASD), Attention Deficit Hyperactivity Disorder (ADHD)	Parents may exhibit vaccine hesitancy due to widespread concerns about a potential association between vaccination and these conditions.
Chronic Condition with Special Vaccination Needs Group	Type 1 Diabetes Mellitus (T1DM), Congenital Heart Disease (CHD	Children with chronic diseases that necessitate additional vaccinations beyond routine childhood immunization.
Chronic Condition with Routine Vaccination Group	Familial Mediterranean Fever (FMF), Congenital Hypothyroidism (CH)	Children who require frequent medical follow-ups but do not need vaccinations beyond the routine schedule.
Healthy Control Group	Healthy children without known comorbidities	Children with no diagnosed chronic conditions or special vaccination requirements.

The sample size was determined based on a review of previously published literature and statistical power analysis [17, 19]. To detect a 20% difference between groups with a 95% confidence interval (CI), 90% power, and 5% margin of error, a minimum of 360 participants (60 per group) was required. The calculation was performed using standard methods for comparing proportions in independent groups, ensuring sufficient statistical power to detect meaningful differences. Given the study’s objective to analyze VH across different parental attitudes, an expanded control group of 585 participants was included to improve the robustness of comparisons. The final sample size accounted for potential dropout or incomplete data, aligning with recommendations from epidemiological research on vaccine acceptance.

The inclusion criteria for the study required participants to have a diagnosis of ASD, ADHD, T1DM, FMF, CHD, or CH. For the control group, eligibility was limited to children aged 6–12 years who presented to the hospital for routine child health follow-up without any acute or chronic disease and voluntarily agreed to participate. Individuals were excluded if they declined to participate for any reason, were outside the specified age range, had a diagnosis other than the specified conditions, lacked a definitive diagnosis, or had an acute or chronic disease in the control group. Additionally, parents who had difficulty understanding or completing the assessment scales were also exclude.

### Survey instruments

All data were collected by the pen and pensil method under the supervision of the research team from only mothers. Three different survey forms were used in the research. The socio-demographic data form used in the study was developed by the researchers by reviewing previous literature (Supplemantary material) [20, 21].

Parental VH was assessed using the 15-item Parent Attitudes about Childhood Vaccines (PACV) survey. The survey was developed by Opel et al. [22] in 2011. The validity and reliability study of the scale for Türkiye was conducted by Çevik et al. in 2020 [23]. The scale comprises 15 items and three sub-dimensions: vaccination behavior, beliefs about vaccine safety and efficacy, general attitudes, and trust. The responses to the items on the scale are of the following types: two questions are closed-ended (yes/no/don’t know), 11 questions are of the 5-point Likert type (strongly agree/agree/unsure/disagree/strongly disagree), and two questions are of the scoring type (from 0 to 10). The total raw score was converted to a scale ranging from 0 to 100, and a parent was defined as hesitant if the score was ≥ 50. If the mother was found to be vaccine hesitant, two further open-ended questions were asked, including the reason for hesitancy and the proposed solution, and the answers were recorded.

The Parental Attitude Research Instrument (PARI) was developed by Schaefer and Bell and translated into Turkish by Le Compte et al. [24]. This 60-item, 4-point Likert scale (4 points I find very appropriate, 1 point I find very inappropriate) aims to measure parents’ relationships with and attitudes towards their children. The scale consists of five factors: (a) Overprotective motherhood (16 items), (b) Democratic attitude and recognition of equality (9 items), (c) Denial of the Housewife Roles (13 items), (d) Marital Conflict (6 items), and (e) Strict discipline (16 items). Separate scores are calculated for each of the subscales. A high score on the subscale indicates that the attitude reflected by that dimension is approved. High scores on Democratic attitude, recognition of equality are considered positive, while high scores on the other factors as negative. The scores of the Parental Attitude Research Instrument (PARI) were divided into quartiles (Table 2), with the lowest segment designated as Q1 and the highest as Q4.

**Table 2:** Parent attitudes about childhood vaccines (PACV) survey and parental attitude research instrument (PARI) subscale scores

	Item no	Mean	SD	Percentiles		
				25	50	75
Parent Attitudes about Childhood Vaccines Survey, converted score	15	32.9	15.2	23.0	30.0	40.0
Parental Attitude Research Instrument Subscale scores						
Over-protective motherhood	16	44.0	9.2	37.0	44.0	50.0
Democratic attitude	9	27.4	3.7	25.0	27.0	30.0
Denial of the housewife roles	13	29.4	6.7	25.0	29.0	34.0
Marital conflict	6	14.1	4.1	11.0	14.0	17.0
Strict discipline	16	37.3	8.4	31.0	36.0	43.0

### Ethical aspect of the research

This study was approved by the Hacettepe University Non-Interventional Clinical Research Ethics Committee (with the 07.09.2021 date and 2021/14–49 number) in accordance with the Declaration of Helsinki. Prior to the study, the parents of all participating children were informed about the study and provided their consent.

### Statistical analysis

The data were evaluated with the IBM SPSS Statistics for Windows, Version 23.0 (Armonk, NY: IBM Corp.). The normality of the data was checked with the Kolmogorov-Smirnov test. If the data met parametric conditions, they were analyzed with independent sample t test for two independent groups and ANOVA for more than two groups. When using ANOVA for comparisons with more than two groups, Tukey’s T2 tests were used for those that met the homogeneity assumption, and Tamhane’s T2 tests were used for those that did not meet the homogeneity assumption, to determine which group was different from the others. If any or all of the assumptions were not met, the Mann Whitney U test was used for two independent groups, and the Kruskal Wallis test was used for more than two independent groups.

The Chi-square test was used to evaluate differences in the frequencies of categorical data. For variables with more than two subgroups, when a significant difference was detected, residual analysis with Bonferroni correction was applied to identify the specific subgroup(s) contributing to the difference.

To determine the relationship between variables, Pearson correlation coefficient was used for parametrics and Spearman correlation coefficient was used for non-parametrics. Multiple logistic regression (Model 1) was conducted using the enter method. The dependent variable was VH, and the independent variables included mother’s education (≥ high school vs. < high school), mother’s employment (unemployed vs. employed), monthly income (middle income vs. low income; high income vs. low income), adverse reaction in child and/or siblings (yes vs. no), COVID-19 vaccination status (reference: both parents vaccinated), ınformation sources for vaccines (reference: no information), child’s disease (ASD vs. control; T1DM vs. control; CHD vs. control). Model 2 included, in addition to the variables from Model 1, the democratic attitude and recognition of equality subscale (Reference: Q1). The Odds ratio and 95% Confidence Interval (CI) were calculated. Variables with *p* < 0.20 in single analyses were taken into further analysis. The error level was taken as 0.05.

## Results

### Study group and sociodemographic characteristics

A total of 1,163 individuals participated in the study, including 88 with ASD, 90 with ADHD, 92 with FMF, 95 with T1DM, 91 with CH, 90 with CHD, and 617 healthy controls. The average age of the children was 8.95 ± 2.36 years, and 54.9% (*n* = 639) were male. All surveys were completed by mothers, of whom 77.6% were unemployed. Nearly half of the participants (49.8%) had a low monthly income. Further sociodemographic details are provided in Table 3.

**Table 3:** Sociodemographic characteristics of the participants and associations with vaccine hesitancy

Sociodemographic data	*N*(%)^a^	Vaccine hesitant (*n*%)^b^	*p* value	PACV survey score (mean ± SD)	*p* value
Overall	1163 (100)	171 (14.7)			
Child age (years)			0.486		0.250
6–9 years	638 (54.9)	98 (15.4)		32.4 ± 15.6	
10–12 years	525 (45.1)	73 (13.9)		33.4 ± 14.7	
Sex			0.623		0.757
Male	639 (54.9)	92 (14.2)		33.0 ± 15.0	
Female	524 (45.1)	80 (15.3)		32.7 ± 15.5	
Mothers’ age			0.938		0.384
< 35 years	439 (37.7)	65 (14.8)		33.4 ± 15.2	
≥ 35 years	724 (62.3)	106 (14.6)		32.6 ± 15.2	
Fathers’ age			0.792		0.574
< 35 years	216 (18.6)	33 (15.3)		33.4 ± 14.8	
≥ 35 years	947 (81.4)	138 (14.6)		32.7 ± 15.3	
Mothers’ education			0.234		0.177
< high school	330 (28.4)	55 (16.7)		33.8 ± 14.9	
≥high school	833 (71.6)	116 (13.9)		32.5 ± 15.3	
Fathers’ education			0.309		0.281
< high school	219 (18.8)	37 (16.9)		33.9 ± 13.7	
≥high school	944 (81.2)	182 (14.2)		32.6 ± 15.5	
Mother’s employment			**0.011**		**0.025**
Housewife	903 (77.6)	145 (16.1)		33.7 ± 15.3	
Employed	219 (22.4)	25 (9.7)		30.5 ± 16.5	
Father’s employment			0.340		0.132
Unemployed	46 (3.9)	10 (21.7)		37.9 ± 14.5	
Education sector	80 (6.9)	15 (18.8)		32.6 ± 16.5	
Health sector	43 (3.7)	7 (6.3)		31.2 ± 2.8	
Others	994 (85.5)	139 (14)		32.7 ± 15	
Family type			0.540		0.283
Nucleer	1010 (86.8)	146 (14.5)		32.7 ± 15.2	
Broken or extended	153 (13.2)	25 (16.3)		34.1 ± 15.1	
Living in					
City Centre	893 (76.8)	131 (14.6)	0.799	32.5 ± 15.4	0.106
District/village	270 (23.2)	41 (15.2)		34.2 ± 14.5	
Household income			**0.031**		**0.004**
Low income	579 (49.8)	101 (17.4)*		34 ± 15.1	
Moderate income	363 (31.2)	43 (11.8)		32.8 ± 13.9	
High income	221 (19)	27 (12.2)		30 ± 17*	
Number of children in the household			0.624		0.907
1	166 (14.3)	27 (16.3)		33.3 ± 16	
2–3	493 (42.4)	67 (13.6)		32.8 ± 14.3	
≥ 4	504 (43.3)	77 (15.3)		33.7 ± 15.8	

^a^column percentage, ^b^row percentage, *post-hoc analyse result, PACV: Parent Attitudes about Childhood Vaccines survey

### Parent attitudes about childhood vaccines (PACV) survey scores

**Fig. 1:**
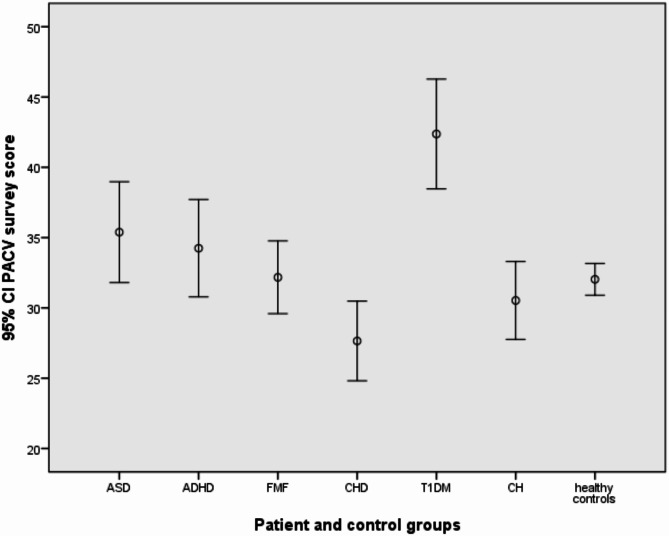
PACV survey average scores of the participants with 95% Confidence Interval [ASD: Autism Spectrum Disorder. ADHD: Attention Deficit and Hyperactivity Disorder. T1DM: Type 1 Diabetes Mellitus. FMF: Familial Mediterranean Fever. CH: Congenital Hypothyroidism. CHD: Congenital Heart Disease. PACV: Parent Attitudes about Childhood Vaccines survey]

The overall mean PACV score was 32.9 ± 15.2, with no significant difference between patient and control groups (*p* = 0.51). The VH rate was 14.7% (*n* = 171), with the highest rate in the T1DM group (33.7%). The group with the highest mean PACV score was T1DM (42.4 ± 19.2), and the lowest was CHD (27.6 ± 13.5). The mean scores of the participants on the PACV survey with a 95% CI are given in Fig. 1. A significant difference was found between the PACV scores of disease groups (*p* < 0.05). Post hoc analysis indicated statistically significant differences between ASD and CHD, as well as between FMF, CHD, ADHD, and T1DM (*p* < 0.05). The total PACV score was statistically significantly lower in employed mothers and high income (*p* = 0.025, *p* = 0.004). The VH rate was statistically significantly higher among housewives and those with low income (*p* = 0.01, *p* = 0.031). Associations between socio-demographic characteristics and VH are presented in Table 3.

At the beginning of the study, we categorized chronic diseases into three main groups: “Chronic Condition (CC) with Potential VH Group (ASD and ADHD)”, “CC with Special Vaccination Needs Group (T1DM and CHD)”, and “CC with Routine Vaccination Group (FMF and CH)”. However, we later observed significant differences within the special vaccination needs group, particularly between T1DM and CHD. Therefore, we evaluated these diseases separately (Tables 1 and 4).

**Table 4:** PACV survey avarege scores and VH rates according to clinical conditions of participants

	Overall, *n*	Vaccine hesitant (%)	PACV survey score (Mean ± SD)
**Chronic Disease Groups**			
ASD^#^	88	19 (21.6)^ab^	35.4 ± 17.0^b^
ADHD^#^	90	17 (18.9)^bc^	34.2 ± 16.5^b^
T1DM^&^	95	32 (33.7)^a^	42.4 ± 19.2^a^
CHD^&^	90	5 (5.6)^d^	27.6 ± 13.5^d^
CH^$^	91	9 (9.9)^cd^	30.5 ± 13.3^bc^
FMF^$^	92	7 (7.6)^d^	32.2 ± 12.5^bc^
		*p* < 0.001	*p* < 0.001
**Chronic Condition type**			
^#^CC with Potential Vaccine Hesitancy Group	178	20.2^a^	34.8 ± 16.7^a^
^&^CC with Special Vaccination Needs Group	182	20.0^a^	35.2 ± 18.2^a^
^$^CC with Routine Vaccination Group	186	8.7^b^	31.4 ± 12.9^b^
		0.003	*P* = 0.043
**Duration of Chronic Condition**,** (years)**			
1	107	20 (18.7)	35.3 ± 16.1
2–3	119	26 (21.8)	34.6 ± 16.5
≥ 4	320	43 (13.4)	33.0 ± 16.0
		0.080	0.362
**Enrolled child without considering siblings**			
Enrolled child having a CC	546	89 (16.3)	33.8 ± 16.1
Healthy child	617	82 (13.3)	32.0 ± 14.3
		0.148	0.051
**Mother’s Status**,** Based on her Children’s Health Condition**^**€**^			
Mothers with a child who has a disease	601	104 (17.3)	34.0 ± 16.0
Mothers with no child who has a disease	562	37 (11.9)	31.7 ± 14.2
		0.010	0.011
**Child’s Health Status**			
Single healthy children	91	12 (13.2)^ab^	33.6 ± 14.9^b^
Two or more children, all healthy	471	55 (11.7)^b^	31.3 ± 14.0^b^
Two or more children, enrolled child is healthy but has a sibling with a disease	55	15 (27.3)^a^	35.5 ± 15.0^ab^
Single child with a disease	77	16 (20.8)^ab^	33.4 ± 17.5^b^
Two or more children, enrolled child has a disease, but the other(s) are healthy	387	56 (14.5)^ab^	32.8 ± 15.9^b^
Two or more children, both/all have a disease	82	17 (20.7)^ab^	38.9 ± 15.3^a^
		0.010	0.001
**Sibling Characteristics**			
No siblings	166	27 (16.3)^ab^	33.3 ± 16.0^a^
Healthy sibling(s)	861	112 (13.0)^a^	32.0 ± 14.9^a^
Sibling(s) with a disease	136	32 (23.5)^b^	37.5 ± 15.3^b^
		0.005	< 0.001
Total	1163	171 (14.7)	32.9 ± 15.0

ASD: Autism Spectrum Disorder, ADHD: Attention Deficit and Hyperactivity Disorder, T1DM: Type 1 Diabetes Mellitus, FMF: Familial Mediterranean Fever, CH: Congenital Hypothyroidism, CHD: Congenital Heart Disease. SD: Standard deviation PACV: Parent Attitudes about Childhood Vaccines survey

^€^Fifty-five children in the control group had a sibling with a disease. Values with different letters (a–d) in the same column for the same variable are statistically significantly different (*p* < 0.05)

The average PACV survey scores and VH rates according to the clinical conditions of the participants are presented in Table 4. The lowest VH rate and PACV score were observed in individuals with a disease duration of four years or more; however, this difference was not statistically significant (*p* > 0.05).

When mothers were categorized into two groups based on whether they had a child with a CC, it was seen that the VH rate and PACV mean scores of mothers with a child with a CC were statistically significantly higher (*p* = 0.010 and *p* = 0.011) compared to those having only healthy children.

When grouped according to the health status of children at home, the lowest VH rate was seen when there were two and/or more healthy children in the household. The highest VH rates were in the group with a single child with a disease, and in the groups with two and/or more children with diseases in the household. Similarly, the highest PACV score was when there were two or more children with diseases, while the lowest PACV mean score was when all children in the household were healthy. The results were statistically significant.

When evaluated according to sibling characteristics, the highest VH rate and PACV score were observed in the sibling(s) with a disease group (*p* = 0.005 and *p* < 0.001, Table 4).

### Parental attitude research instrument (PARI) subscale scores

When VH rates were examined according to parental attitudes, no relationship was found between VH rates and the quartiles of overprotective motherhood, denial of the housewife roles, and strict discipline subscales (*p* = 0.212, *p* = 0.780, and *p* = 0.529, respectively). However, as the democratic attitude subscale score increased, VH rate and the mean PACV score decreased statistically significantly (*p* = 0.010 and *p* = 0.002, respectively). In the strict discipline subscale, while no significant relationship was found between the scale score and VH rate (*p* = 0.529), an increase in strict discipline was associated with a significant increase in the mean PACV score (*p* = 0.037). Regarding the Marital Conflict subscale, individuals in the lowest quartile (Q1) had a significantly lower VH rate and PACV score compared to the higher quartiles (*p* = 0.029 and *p* = 0.008, respectively). A comparison of VH frequency and PACV survey scores across quartiles of PARI subscale scores is presented in Table 5.

**Table 5:** Comparison of VH frequency and PACV survey scores across quartiles of PARI subscale scores

PARI	*N*(%)^a^	Vaccine hesitant (*n*%)^b^	*p* value	PACV survey score (mean ± SD)	*p* value
**Over-protective motherhood**			0.212		0.118
Q1	291 (25.0)	40 (13.7)		31.6 ± 17.3^a^	
Q2	300 (25.8)	45 (15.0)		31.5 ± 14.4^a^	
Q3	288 (24.8)	52 (18.1)		34.9 ± 15.2^b^	
Q4	284 (24.4)	34 (12.0)		33.3 ± 14.0^ab^	
**Democratic attitude**,** recognition of equality**			**0.010**		**0.002**
Q1	280 (24.1)	**57 (20.4)** ^**a**^		35.3 ± 16.3^a^	
Q2	311 (26.7)	**47(15.1)** ^**ab**^		33.4 ± 15.8^ab^	
Q3	320 (27.5)	**36 (11.3)** ^**b**^		32.0 ± 13.9^bc^	
Q4	252 (21.7)	**31 (12.3)** ^**b**^		30.5 ± 14.4^c^	
**Denial of the housewife roles**			0.780		0.338
Q1	281 (24.2)	32 (11.4)		32.0 ± 15.5	
Q2	256 (22.0)	32 (12.5)		32.0 ± 15.2	
Q3	309 (26.6)	56 (18.1)		33.9 ± 15.4	
Q4	317 (27.3)	51 (16.1)		33.3 ± 14.7	
**Marital conflict**			**0.029**		**0.008**
Q1	328 (28.2)	33 (10.1)^a^		30.5 ± 14.2^a^	
Q2	309 (26.6)	48 (15.5)^b^		33.6 ± 15.3^b^	
Q3	265 (22.8)	49 (18.5)^b^		34.6 ± 16.0^b^	
Q4	261 (22.4)	41 (15.7)^b^		33.2 ± 15.1^b^	
**Strict discipline**			0.529		**0.037**
Q1	310 (26.7)	40 (12.9)		31.2 ± 17.0^a^	
Q2	280 (24.1)	38 (13.6)		32.2 ± 14.8^ab^	
Q3	277 (23.8)	46 (16.6)		33.9 ± 14.7^b^	
Q4	296 (25.5)	47 (15.9)		34.3 ± 13.9^b^	

Q: quartile. Values with different letters (a, b, c) in the same column for the same variable are statistically significantly different ( *p* < 0.05)

### Vaccine adverse effects and vaccine hesitancy

The overall incidence of vaccine-related adverse effects in enrolled children was 4.1%. The occurrence of vaccine adverse effects in either the study participant child and their sibling(s) was 5.2%. Parents of children who experienced vaccine adverse events had significantly higher PACV scores (40.8 ± 21.9) compared to those whose children did not experience such events (32.4 ± 14.6, *p* = 0.005). The mean PACV score was statistically significantly lower in fully vaccinated child and fully vaccinated sibling groups. The VH rate in these participants was also significantly lower than in unvaccinated- incompletely vaccinated participants (*p* < 0.05).

### Vaccine hesitancy and associated factors

Regarding parental COVID-19 vaccination status, children whose both parents were vaccinated had the lowest VH rate (11.1%) and the lowest PACV score (31.0 ± 13.9), whereas those with both parents unvaccinated had the highest VH rate (31.1%) and PACV score (42.8 ± 19.3) (*p* < 0.001 for both, Table 6). Fathers who had received an influenza vaccination also exhibited lower PACV scores and VH rates (*p* < 0.001).

**Table 6:** Vaccination status of the family mambers, information sources for vaccines, and VH

Vaccination status	*N* (%)^a^	Vaccine hesitant (*n*%)^b^	*p* value	PACV score (mean ± SD)	*p* value
Child			**< 0.001**		**0.003**
Fully vaccinated	1151 (99)	164 (14.2)		32.6 ± 14.8	
Unvaccinated/Incompletely vaccinated	12 (1)	7 (58.3)		60.8 ± 26.3	
Siblings of the child (*n* = 998)			**< 0.001**		**< 0.001**
Fully vaccinated	990 (85.1)	138 (13.9)		32.5 ± 14.7	
Unvaccinated/Incompletely vaccinated	8 (0.7)	7 (87.5)		69.5 ± 21.1	
Adverse event in child’s vaccination			**< 0.001**		**< 0.001**
Yes	48 (4.1)	19 (39.6)		32.3 ± 14.6	
No	1115 (95.9)	152 (13.6)		45 ± 14.6	
Adverse event in siblings			**0.017**		0.290
Yes	23 (2.2)	8 (30.8)		32.7 ± 14.8	
No	972 (83.6)	137 (14.1)		37.6 ± 23.4	
Adverse event in child and/or siblings			**< 0.001**		**0.005**
Yes	60 (5.2)	40 (66.7)		40.8 ± 21.9	
No	1103 (94.8)	952 (86.3)		32.4 ± 14.6	
COVID-19 Vaccination status			**< 0.001**		**< 0.001**
Both parents vaccinated	888 (76.4)	99 (11.1)		31.0 ± 13.9	
Only mother vaccinated	86 (7.4)	20 (23.3)		35.3 ± 14.3	
Only father vaccinated	83 (7.1)	19 (22.9)		37.3 ± 17.5	
Both parents unvaccinated	106 (9.1)	33 (31.1)		42.8 ± 19.3	
Tetanus Vaccine			0.979		0.903
Both parents vaccinated	146 (12.6)	20 (13.7)		32.6 ± 15.3	
Only mother vaccinated	214 (18.4)	31 (14.5)		33.1 ± 15.2	
Only father vaccinated	90 (7.7)	14 (15.6)		33.9 ± 15.7	
Both parents unvaccinated	713 (61.3)	106 (14.9)		32.7 ± 15.1	
Influenza vaccine			0.133		**0.002**
Both parents vaccinated	22 (1.9)	0 (0.0)		25.3 ± 11.4	
Only mother vaccinated	18 (1.5)	4 (22.2)		32.3 ± 20.5	
Only father vaccinated	25 (2.1)	2 (8.0)		24.0 ± 15.1	
Both parents unvaccinated	1098 (94.4)	165 (15.0)		33.2 ± 15.1	
**Information sources for vaccines**					
Health personnel			**< 0.001**		**< 0.001**
Yes	1067 (91.7)	135 (12.7)		32.0 ± 14.4	
No	96 (8.3)	36 (37.5)		42.6 ± 19.7	
Books and journals			**< 0.001**		**0.022**
Yes	86 (7.4)	27 (31.4)		37.6 ± 14.7	
No	1077 (92.6)	144 (13.4)		32.5 ± 14.7	
Internet web sites			**< 0.001**		**< 0.001**
Yes	182 (15.6)	47 (25.8)		37.1 ± 18.7	
No	981 (84.4)	124 (12.6)		32.1 ± 14.4	
Facebook, Instagram			**< 0.001**		**0.029**
Yes	30 (2.6)	13 (43.3)		42.0 ± 22.2	
No	1133 (97.4)	158 (13.9)		32.6 ± 14.9	
Family elder or opinion leader			0.392		0.064
Yes	24 (2.1)	5 (20.8)		38.5 ± 17.9	
No	1139 (97.9)	166 (14.6)		32.7 ± 15.1	
Others			0.784		0.630
Yes	24 (2.1)	4 (16.7)		31.4 ± 16.7	
No	1139 (97.9)	167 (14.7)		32.9 ± 15.2	

^a^Row percentage ^b^column percentage, SD: Standard deviation PACV: Parent Attitudes about Childhood Vaccines survey

The VH percentage and mean PACV score were significantly lower among parents who received vaccine information from healthcare personnel (*p* < 0.001 for both). Conversely, parents who obtained vaccine information from books and journals (*p* < 0.001 and *p* = 0.022), websites (*p* < 0.001 for both), or social media platforms (*p* < 0.001 and *p* = 0.029) had significantly higher levels of VH and PACV scores. No significant relationship was found between VH rate and receiving vaccine information from family elders, opinion leaders, or other sources (*p* > 0.05, Table 6).

### Evaluation of incomplete vaccinated children and their siblings

A total of 12 incompletely vaccinated children and 8 incompletely vaccinated siblings were identified (Table 7). Among these cases, 3 had T1DM, 1 had ASD, 2 had FMF, 2 had CHD, and 4 were healthy controls. It is important to note that not all cases of incomplete vaccination necessarily reflect VH, as vaccinations may have been delayed due to underlying CC or ongoing treatments.

**Table 7:** Incomplete vaccinated children and/or their siblings

Age, Gender	Enrolled child				Sibling				Parents COVID-19 vaccination status (Mother/Father)
	Health status	Vaccine status	VAE		Health status	Vaccine status	VAE		
6, F	T1DM	Incomplete	√		Healthy	Incomplete	-		x x
8, F	T1DM	Incomplete	x		T1DM	Incomplete	-		x x
8, M	T1DM	Incomplete	x		Celiac Disease	Incomplete	-		x x
12, M	T1DM	Fully	x		Healthy	Incomplete	-		x x
12, M	T1DM	Fully	x		Metabolic Disease	Incomplete	-		x x
8, M	ASD	Incomplete	x		Healthy	Fully	-		√ √
12, M	ASD	Fully	x		Healthy	Incomplete	-		√ √
12, F	FMF	Incomplete	x		FMF	Fully	-		√ √
11, M	FMF	Incomplete	x		Healthy	Fully	-		√ √
12, F	CHD	Incomplete	x		FMF	Fully	-		√ √
10, F	CHD	Incomplete	x	-		-			x √
7, F	Healthy	Incomplete	x		Healthy	Fully	-		√ √
11, M	Healthy	Fully	x		Romotologic Disease	Incomplete	-		√ √
6, M	Healthy	Incomplete	√		Healthy	Incomplete	-		√ √
12, F	Healthy	Incomplete	√	-		-			x x
6, M	Healthy	Incomplete	x	-		-			√ x

VAE: Vaccine Adverse Event, M: Male, F: Female, √: presence, x:absence, ASD: Autism Spectrum Disorder, T1DM: Type 1 Diabetes Mellitus, FMF: Familial Mediterranean Fever, CHD: Congenital Heart Disease

The prevalence of incomplete vaccination in the T1DM group was 5.3%. Among the three incompletely vaccinated T1DM patients, one had a sibling with celiac disease, one had a sibling with T1DM, and one had a healthy sibling, all of whom were also incompletely vaccinated. None of the parents of these T1DM patients had received the COVID-19 vaccine. In the ASD group, the sibling of an incompletely vaccinated ASD patient was fully vaccinated, while the sibling of a fully vaccinated ASD patient was incompletely vaccinated. Notably, the parents of both ASD cases were vaccinated against COVID-19. Among the FMF patients with incomplete vaccinations, both had fully vaccinated siblings and vaccinated parents, suggesting that vaccination delays may have been due to the CC rather than vaccine refusal. In the CHD group, one child with incomplete vaccination had a fully vaccinated sibling with FMF and both parents vaccinated against COVID-19. The other CHD patient had no siblings, and only one parent was vaccinated.

There was a vaccination problem in 5 healthy cases (0.8%). Among the healthy controls with incomplete vaccinations, two had no siblings; one of them had unvaccinated parents. Another healthy control and her sibling both had incomplete vaccinations, but their parents were vaccinated against COVID-19. One healthy control with vaccinated parents had a sibling with a rheumatological disease who was incompletely vaccinated. Additionally, a healthy control with both vaccinated parents and sibling also had incomplete vaccinations.

### Parents’ comments and suggestions about vaccine hesitancy

The most common reason for VH was ‘concerns that vaccine ingredients cause diseases’ with a rate of 40.2%, while the most common proposed solution was ‘more information about vaccines from health professionals’ with a rate of 33.3%. While the most common reason for VH was similar across all three groups, it is notable that the most common proposed solution in the ASD group was the ‘production of vaccines with safe ingredients’. The 3 most common reasons for vaccine hesitant parents and the 3 best proposed solutions in the all vaccine hesitants, ASD and T1DM groups are presented in Table 8.

**Table 8:** The three most common reasons for vaccine hesitancy and the three most effective solutions

	The 3 most common reasons for vaccine hesitancy (%)	%
ASD	Concerns that vaccine ingredients cause diseases Concerns that vaccines do not protect sufficiently from diseases Fear of adverse effects Concerns about vaccine application procedures (lack of oversight, excessive number of applications, etc.)	69.2 15.4 7.7 7.7
T1DM	Concerns that vaccine ingredients cause diseases Concerns about vaccine application procedures (lack of oversight, excessive number of applications, etc.) Fear of adverse effects	57.7 15.4 7.7
All vaccine hesitants	Concerns that vaccine ingredients cause diseases Fear of adverse effects Concerns about vaccine application procedures (lack of oversight, excessive number of applications, etc.)	40.2 22.5 13.7

	The 3 most common proposed solutions to vaccine hesitancy (%)	%
ASD	Producing vaccines with safer ingredients More information about vaccines from health professionals Tighter monitoring of vaccines No vaccinations be administered	41.7 16.7 16.7 16.7
T1DM	More information about vaccines from health professionals No vaccinations be administered Tighter monitoring of vaccines Producing vaccines with safer ingredients	29.2 29.2 12.5 12.5
All vaccine hesitants	More information about vaccines from health professionals Tighter monitoring of vaccines Producing vaccines with safer ingredients No vaccinations be administered	33.3 14.4 14.4 14.4

ASD: Autism Spectrum Disorder, T1DM: Type 1 Diabetes Mellitus

### Factors associated with vaccine hesitancy: binary logistic regression analysis

Parents of children with T1DM had a 3.31 times higher odds of VH compared to the control group (95% CI: 2.04–5.38), while parents of children with ASD had a 1.8 times higher odds (95% CI: 1.03–3.14). In contrast, parents of children with CHD had a lower odds of VH compared to controls (OR: 0.38, 95% CI: 0.15–0.97, Table 9).

**Table 9:** The relationship between the child’s disease and vaccine hesitancy, logistic regression analysis

Child’s disease	OR	95% CI	*p* value
T1DM vs. control	3.31	2.04–5.38	**< 0.001**
ASD vs. control	1.80	1.03–3.14	**0.040**
ADHD vs. control	1.52	0.85–2.71	0.155
CH vs. control	0.72	0.35–1.48	0.368
FMF vs. control	0.54	0.24–1.20	0.130
CHD vs. control	0.38	0.15–0.97	**0.044**

OR: odds ratio, %95 CI: %95 confidence interval, ASD: Autism Spectrum Disorder, ADHD: Attention Deficit and Hyperactivity Disorder, T1DM: Type 1 Diabetes Mellitus, FMF: Familial Mediterranean Fever, CH: Congenital Hypothyroidism, CHD: Congenital Heart Disease

The analysis revealed that several factors related to the mother and the child, including monthly income, the occurrence of side effects in the child or their sibling, the parents’ status of receiving the COVID-19 vaccine, the child’s diagnosis, the source of information abaout vaccines, had a statistically significant association with the risk of VH. It was observed that a monthly income of twice the minimum wage was associated with a lower odds of VH compared to the minimum wage [OR (95% CI): 0.70 (0.46–1.08)]. The occurrence of a vaccine adverse effect in a child or sibling was associated with a 2.5-fold higher likelihood of VH (95% CI: 1.35–4.94). Having a sibling with a disease increased the risk of VH by 2.34 times compared to having a healthy sibling (95% CI: 1.40–3.89). The odds of VH were found to be 2.34 times higher when neither parent was vaccinated against COVID-19 compared to when both parents were vaccinated (95% CI: 1.31–4.20). Considering the sources of information about vaccines, the odds was lower for those who received information from healthcare personnel [OR (95% CI): 0.48 (0.25–0.93)], while the odds of VH was found to be two times higher for those who received information from the internet (95% CI: 1.26–3.07) (Model 1). When parental attitudes were added to the model, significantly lower VH was found at values ​​above the third quartile of the democratic attitude and equality recognition subscale compared to the first quartile [OR (95% CI): 0.53 (0.32–0.87)]. In the marital conflict subscale, VH was found to be twice as high at values ​​above the third quartile compared to the first quartile, and it was statistically significant (95% CI: 1.17–3.38). (Model 2). In model 1, the odds were found to be 0.39 times lower in FMF patients compared to controls, 0.31 times lower in CHD patients compared to controls, while the odds were found to be 2.77 times higher in T1DM patients compared to controls. In model 2, in which parental attitudes were included, FMF lost statistical significance, while the T1DM odds value increased to 3.02 (95% CI:1.76–5.19). The results of the multiple logistic regression analysis are presented in Table 10.

**Table 10:** Comparison of the relationship between family and child characteristics, parental attitudes and vaccine hesitancy, multiple logistic regression analysis (Model 1 and model 2)

	Model 1			Model 2	
	AOR [%95 CI]	*p* value		AOR [%95 CI]	*p* value
**Mothers’ education**					
≥ high school vs. < high school	0.81 [0.53–1.22]	0.310		0.80 [0.53–1.23]	0.315
**Mothers Employment**					
Employed vs. unemployed	0.70 [0.39–1.26]	0.232		0.67 [0.36–1.23]	0.191
**Monthly income**		0.254			0.215
Middle income vs. low income	0.70 [0.46–1.08]	0.104		0.68 [0.44–1.05]	0.080
High income vs. low income	0.76 [0.4–1.43]	0.395		0.80 [0.42–1.51]	0.485
**COVID-19 Vaccination status**		<0.001			<0.001
Only mother vs. both vaccinated	**2.98 [1.78–4.98]**	**<0.001**		**2.86 [1.68–4.84]**	**<0.001**
Only father vs. both vaccinated	**2.30 [1.26–4.21]**	**0.007**		**2.47 [1.34–4.55]**	**0.004**
Neither vs. both vaccinated	**2.34 [1.31–4.20]**	**0.004**		**2.48 [1.37–4.50]**	**0.003**
**Information sources for vaccines**					
Health personnel vs. no information	**0.48 [0.25–0.93]**	**0.031**		**0.47 [0.24–0.91]**	**0.026**
Books and journals vs. no information	1.63 [0.78–3.4]	0.196		1.5 [0.71–3.18]	0.292
Internet vs. no information	**1.97 [1.26–3.07]**	**0.003**		**2.04 [1.3–3.21]**	**0.002**
**Adverse reaction in child and/or siblings**					
Yes vs. no	**2.58 [1.35–4.94]**	**0.004**		**2.44 [1.26–4.73]**	**0.008**
**Child disease**		<0.001			<0.001
ASD vs. control	1.74 [0.95–3.19]	0.073		1.68 [0.92–3.1]	0.094
ADHD vs. control	1.28 [0.68–2.39]	0.447		1.32 [0.7–2.48]	0.392
FMF vs. control	**0.39 [0.16–0.95]**	**0.039**		0.42 [0.17–1.01]	0.053
CHD vs. control	**0.31 [0.12–0.82]**	**0.018**		**0.34 [0.13–0.91]**	**0.032**
T1DM vs. control	**2.77 [1.63–4.69]**	**<0.001**		**3.02 [1.76–5.19]**	**<0.001**
CH vs. control	0.72 [0.33–1.54]	0.391		0.74 [0.34–1.62]	0.450
**Siblings**		**0.002**			**0.005**
Sibling(s) with a disease vs. Healthy sibling(s)	**2.34 [1.40–3.89]**	**0.001**		**2.27 [1.35–3.83]**	**0.002**
No sibling vs. Healthy sibling(s)	1.59 [0.95–2.65]	0.076		1.47 [0.87–2.48]	0.150
**Over Protective Motherhood**					0.286
Q2 vs. Q1				1.07 [0.64–1.81]	0.790
Q3 vs. Q1				1.05 [0.62–1.79]	0.851
Q4 vs. Q1				0.66 [0.36–1.2]	0.173
**Democratic Attitude**					0.082
Q2 vs. Q1				0.68 [0.42–1.1]	0.112
Q3 vs. Q1				**0.53 [0.32–0.87]**	**0.013**
Q4 vs. Q1				0.65 [0.38–1.09]	0.103
**Marital Conflict**					0.090
Q2 vs. Q1				1.55 [0.92–2.59]	0.100
Q3 vs. Q1				**1.99 [1.17–3.38]**	**0.012**
Q4 vs. Q1				1.61 [0.91–2.83]	0.100
Constant	0.52	0.113		0.53	0.208

AOR: adjusted odds ratio. 95% CI: 95% confidence interval. ASD: Autism Spectrum Disorder, ADHD: Attention Deficit and Hyperactivity Disorder, T1DM: Type 1 Diabetes Mellitus, FMF: Familial Mediterranean Fever, CH: Congenital Hypothyroidism, CHD: Congenital Heart Disease; Q: quartile

## Discussion

This study included 1,163 participants: 546 patients from six different disease groups and 617 healthy controls. Our research provides valuable information on VH and vaccination behaviors among parents of children with chronic diseases in Türkiye. We also attempted to identify the underlying factors influencing these behaviors and assessed parental attitudes. By examining the relative determinants of VH, this study provides data on how chronic diseases in children may shape parental vaccination decisions.

Parent Attitudes about Childhood Vaccines (PACV) survey is a validated tool for identifying vaccine-hesitant parents (VHPs). Studies using this survey report VH rates between 6.7% and 34.7% in general populations [17, 23, 25–30]. The prevalence of VH appears to differ between countries due to cultural and socioeconomic differences. In our study, we found the VH rate 14.7%. It is also noteworthy that the high VH rate is observed in healthy controls (13.3%). In their study of 575 participants, which included cases of both vaccine acceptance and refusal, Bianco et al. found that the VH rate was 7.7% by PACV survey [31].

Vaccine hesitancy was significantly higher among housewives and families with lower monthly income (*p* = 0.01 and *p* = 0.031). Vaccine refusal based on socio-demographic characteristics is a controversial issue in the literature. In a similar study we conducted previously, we found that vaccine acceptance was low among mothers with below than a high school education and among unemployed parents [20]. Although there are studies reported that low educational and socioeconomic levels increase VH and VR [32, 33], there are also studies that argue the opposite [34]. In their study conducted in Indonesia, Yufika et al. observed that VH was more prevalent among mothers, parents with a younger age profile, and individuals with lower levels of education [29]. In their study conducted in the United Arab Emirates, Alsuwaidi et al. found no evidence that the education level, income level, or age of the parents were associated with VH. An intriguing observation was that VH was prevalent among divorced parents [35]. The relationship between sociodemographic characteristics and VH is highly variable due to the influence of different cultures and different populations living in different societies.

Children with chronic diseases are at greater risk of complications from VPDs, so it is very important that they should be fully vaccinated according to their age [36]. Especially, children with diabetes are vulnerable to infections due to the direct effects of hyperglycemia, DM-related immune dysregulation (decreased phagocytic activity, neutrophil chemotaxis, and T-cell function), and it is important for them to be fully vaccinated to protect them from VPDs [37].

At the beginning of our study, we classified the patient groups into three categories based on their clinical conditions: potential VH group, special vaccination needs group, and routine vaccination group. While analyzing the results, we observed a significant difference between T1DM and CHD within the special vaccination needs group. Given the substantial disparities between these conditions, it would have been inappropriate to evaluate them together. Therefore, we decided to analyze each disease separately and discuss the findings in detail, allowing for a more accurate understanding of VH and vaccination needs within these distinct groups. Considering the differing immunological and medical challenges faced by children with T1DM and CHD, separate analyses provide a more nuanced perspective on vaccination barriers and facilitators, ultimately leading to more targeted public health interventions.

In our study, although it is a very critical group to be vaccinated, it was observed that 33.7% of the parents of children with T1DM were vaccine hesitant, and 5.26% of the parents of children with T1DM refused vaccination in their sick children or siblings. The majority of these VHPs believed that T1DM developed after the measles, mumps, and rubella (MMR) vaccine. Few studies exist on vaccine acceptance among parents of children with T1DM. In their epidemiological cohort study, Glanz MJ. et al. [38] employed three vaccination criteria (average number of vaccinated days, cumulative aluminum, and cumulative antigen exposure) to evaluate the relationships between the current childhood vaccination program in the USA and T1DM. The results demonstrated that the recommended vaccination program did not increase the risk of developing T1DM. In their study investigating the opposition to the COVID-19 vaccine among parents of children with T1DM, Çelik and Doğan found that approximately half of the parents (46.1%) expressed hesitancy about vaccinating their children. Additionally, 21.6% of parents had not been vaccinated against COVID-19 [39]. Napolitano et al. found a 23.7% VH rate among parents of children with chronic diseases by applying the PACV survey, with 27.7% among T1DM parents [17]. In our study, this rate was found to be 33.7%. This group of parents should be considered to be at high risk for VH and VR, and the necessary precautions should be taken as soon as possible. One of the most important gains of our study is that we detected this. Furthermore, Napolitano et al. identified the presence of VH in parents of children with additional endocrinological, rheumatological, and hematological-oncological disorders. However, the number of cases remained below the desired level [17]. In our study, the VH rate in the CH, CHD, and FMF groups was found to be lower than even healthy controls. The age at which the cases were diagnosed and the family’s interpretation of this may also be related to VH. Since CHD is congenital and families have regular follow-ups without associating it with environmental factors, it may be thought that VH did not occur. Since T1DM develops later, families may be associating the disease with environmental factors and vaccines. In our study, we found that the type of CC as well as the duration of the CC had an effect on VH. As the duration of the CC increased, VH decreased.

Despite all the scientific evidence, there is no relationship between ASD and vaccines, VH is more common among parents of children with ASD [11]. Although children diagnosed with ASD are vaccinated regularly until the age of two, it is known that vaccination rates subsequently decrease due to their parents’ concerns about the potential link between vaccines and ASD [12]. In our study, VH among parents of children with ASD was 21.6%. In a similar study conducted by Goin-Kochel et al. [40] with 225 participants using the PACV survey, the VH rate in parents of children with ASD was found to be 28.8%. In their study evaluating VH in ASD, non-ASD developmental disorders, rheumatological conditions, and the general pediatric population, by PACV survey, Sahni et al. [10] found that the overall VH rate was 19.9%. They also observed that parents of children with ASD reported the highest VH rates (29.5%). In another study, the rate of VH in parents of children with ASD and non-ASD neurodevelopmental defects was 23.6%, the rate was very high in parents with ASD [41]. Our findings also indicate elevated VH in this group.

In studies investigating parents’ beliefs about the causes of their child’s ASD; genetics, the child’s brain structure, and the will of God emerge as the most common beliefs [10, 42]. Although some studies have indicated that the perception of a causal relationship between vaccines and autism is relatively low [42], there are also studies involving parents who associate vaccines with autism at a high rate [40]. Bağ and Güney found that high income, the use of social media as the primary source of information, and the defisit of regular well-child visits; are risk factors for the development of VR among parents of children with ASD [43]. It is important to note that young siblings of children with ASD are at risk of unvaccination [12, 44, 45]. In our study, only one of the siblings of children with ASD had VR (1.3%). However, the VR rate in siblings of children with T1DM, the group in which we found the highest VH rate, was 5.8%. The healthcare professionals should be aware of this risk, and that the vaccination uptake of younger siblings of children not only with ASD but also T1DM and other chronical diseases are evaluated with greater scrutiny.

The most common reasons for VH in our study were concerns about vaccine ingredients causing diseases (40.2%) and fear of adverse effects (22.5%). Similarly, a study from our country identified distrust in vaccines and beliefs about their potential danger to children as key factors influencing VR [46]. In the study conducted on VR cases in Türkiye in 2016–2017, concerns about harmfulness (infertility, disability, autism), observed or heard adverse effects of vaccines and religious beliefs against vaccination were the three most common reasons [15]. In a study that evaluated VHPs with and without children with ASD, parental concerns about vaccines, and vaccine risk perception had contributed to the decision to vaccinate [47]. The occurrence of adverse effects subsequent to vaccination is also regarded as a contributing factor to VH. In a retrospective study conducted on parents of children with ASD, a higher prevalence of adverse reactions was observed in both their children with ASD and their siblings (22.6% and 6.9%) [45]. In our study, the overall vaccine adverse reaction rate was 5.2%. The rate was 6.8% in children with ASD and 1.13% in their siblings. The average PACV survey score of the parents of children who experienced vaccine adverse effects was statistically significantly higher than those who did not experience adverse effects (*p* = 0.005). The group with the highest vaccine adverse effects was T1DM (9.5%). One of the underlying reasons for the high VH in this group and ASD group may be the adverse effect rates. It may be beneficial to provide families with information regarding potential adverse effects, their prevalence, and recommended actions in the event of such effects.

Sahni et al. [10] in their study on ASD, rheumatological, and neurodevelopmental patient groups, found that parents did not associate the conditions that caused their child’s disease with their opposition to vaccination. In contrast, the results of our study indicated that 5.9% of parents associated their child’s illness with vaccines. This was especially evident in parents of children diagnosed with T1DM and ASD, and as expected, PACV scale scores were also high. To solve VH and VR, it is important to inform parents in detail about the pathophysiology of the diseases and to clarify that these cannot be attributed to vaccines. Health professionals have important duties in this regard.

A growing number of individuals are relying on the internet and social media platforms such as Facebook, Instagram, and X as their primary source of information regarding health protection and vaccines. VH and VR are increasing worldwide due to this disinformation and fake news on social media [48]. A study conducted by Topçu et al. revealed that approximately one-third of cases of VR received their information about vaccines from social media [46]. In another study where the VH rate was high, the Internet was the main source of information on vaccination [30]. As observed in our study and in the existing literature, the majority of VHPs utilized the internet and social media as their primary sources of information regarding vaccines. In our study, parents’ primary sources of information about vaccines were identified as health proffessionals. A review of the literature revealed that in the majority of studies reviewed, parents’ sources of information about vaccination were health professionals [18]. The VH rate was also statistically significantly lower in individuals who received information from health professionals (*p* < 0.001). It is imperative that health professionals dedicate a greater proportion of their time to accurately informing parents about the benefits and necessity of vaccines. Healthcare personnel are the ‘key people’ to provide the right information, remove doubts, and increase confidence in vaccines among vaccine hesitant families. To overcome VH, adequate information, effective communication, and trust between health professionals and parents should be provided [15].

Our study observed an inverse relationship between democratic parental attitudes and VH, suggesting that higher democratic attitudes may reduce VH by fostering open-mindedness and critical thinking. Additionally, personal challenges, such as marital conflicts, can heighten parental scrutiny of vaccines, increasing hesitation and uncertainty due to the stress they create, which may amplify vaccine skepticism. The influence of family dynamics on VH underscores the need to incorporate parental well-being and household stressors into public health strategies aimed at improving vaccine acceptance. These findings highlight the importance of a comprehensive approach, integrating educational interventions with consideration of emotional and social factors that shape parental decision-making. Targeted interventions that address both informational gaps and the broader psychosocial context are essential for effectively reducing VH and improving vaccination rates.

Giambi et al. [49] reported a VH rate of 15.6% among parents of children aged 16–36 months, identifying three key risk factors: exposure to parents who experienced severe vaccine adverse effects, lack of pediatrician recommendations for full vaccination, and reliance on alternative medicine. Another study found that increasing economic distress and situations where parents did not make decisions together increased the risk of VH. Experiencing vaccine adverse effects increased VH by 3.36 times and, in severe cases, by 8.65 times. Parents’ age and education level did not influence the risk of VH [32]. In our study, experiencing vaccine adverse events increased the risk of VH by 2.58 times. Similar to our study, experiencing vaccine side effects has been identified as a serious risk factor for VH in many studies conducted to date. Dube et al. [50]. also emphasize that vaccine adverse events have a significant impact on parents’ attitudes towards vaccination and that this may constitute a barrier to vaccine acceptance. Effective management of vaccine adverse events by healthcare professionals is crucial. Addressing misinformation and fears about vaccine adverse events is an important strategy to combat VH. Informing families before vaccination about the possibility of side effects, their frequency, and what needs to be done may help reduce VH.

When studies evaluating VH in parents of children with chronic diseases are examined, Sahni et al. [10] found that, compared to children diagnosed with ASD, the odds of VH were lower in non-ASD neurodevelopmental disease, rheumatological disease, and control groups. Bonsu et al. [41] found that being the parent of a child diagnosed with ASD increased the odds of VH by 3.7 times compared to children with neurodevelopmental diseases without ASD. We found that the risk of VH increased 3.31 times in parents of children diagnosed with T1DM and 1.8 times in those diagnosed with ASD. The risk of VH was 62% lower in parents of children diagnosed with CHD. These findings indicate that the child’s illness has a significant impact on the risk of VH in parents. Our results highlight an elevated VH risk among parents of children with ASD, which aligns with previous studies. Notably, we also identified parents of children with T1DM as having the highest VH risk, contributing new insights to the field. This emphasizes the need for targeted interventions for these high-risk groups to address VH effectively.

Regarding COVID-19 VH, a study on parents of children with neurodevelopmental disorders found that those who remained unvaccinated had 12.14 times higher odds of VH [51]. In the multiple logistic regression analysis model of Temsah et al. [52] study, it was determined that parents’ having received the COVID-19 vaccine, being older, and having a low education level positively increased childhood vaccination. In a study from Türkiye, Durmaz et al. [53] found that parents who were hesitant about childhood vaccinations had lower positive attitudes toward the COVID-19 vaccines. Consistently, our study observed increased VH when one or both parents had not received the COVID-19 vaccine, highlighting the broader societal impact of COVID-19 vaccine distrust on routine immunization.

In this study, VH was assessed using two approaches: a PACV scale threshold of ≥ 50 and the total PACV score. The analysis indicated that in certain groups, despite elevated total PACV scores, the proportion of individuals exceeding the threshold of 50 was not markedly high. This finding suggests that while the total PACV score may indicate an overall increase in VH tendencies, the proportion of individuals exceeding the VH threshold (≥ 50) does not always rise in parallel. This implies that certain factors might contribute to a general reluctance toward vaccination without necessarily leading to outright hesitancy. In other words, some parents may have moderate concerns reflected in higher PACV scores, but these concerns may not be strong enough to classify them as vaccine-hesitant based on the predefined cutoff. This distinction is important in interpreting VH, as interventions targeting vaccine confidence should not only focus on those who surpass the threshold but also address the broader spectrum of hesitancy levels.

When the analysis included healthy control children with siblings affected by CC and mothers were categorized based on whether they had a child with a CC, it was found that mothers of chronically ill children exhibited significantly higher VH rates. These findings underscore the importance of assessing VH at the household level rather than focusing solely on individual children, as the presence of chronic illness within the family may have a broader impact on parental vaccine attitudes.

Among the children diagnosed with diabetes, three had incomplete vaccinations, and five of their siblings were also incompletely vaccinated. Furthermore, it was noted that the parents of these children did not receive the COVID-19 vaccine during the pandemic. This observation highlights that individuals with diabetes, as well as their families, constitute a high-risk group for both VH and VR. Consequently, it is essential to develop targeted educational programs for individuals with diabetes and to provide appropriate support to their families. These results emphasize that siblings of children with CC are also at risk of incomplete vaccination, underlining the need for a comprehensive family-based approach in vaccination strategies. Moreover, one healthy control case experienced an adverse effect following vaccination, leading to both the individual and their sibling having incomplete vaccinations. This finding suggests that adverse effects, even in isolated cases, can influence vaccination attitudes and decisions at the family level. Therefore, addressing VH effectively requires not only dispelling misconceptions about vaccines but also ensuring that families receive adequate information and support in managing potential adverse effects.

Vaccine hesitancy is a complex issue affected by many individual, socioeconomic, political, cultural, and religious factors. The reasons should be evaluated with a holistic approach, and the necessary strategic interventions should be made with the results obtained from the analysis of the reasons. Our findings highlight the need for targeted interventions addressing VH, particularly among parents of children with ASD and T1DM, by enhancing trust in healthcare providers, combating misinformation, and improving communication about vaccine safety and efficacy.

### Strengths and limitations

Our study is the largest study in the literature evaluating parents’ VH with different chronic disease groups and healthy controls. The presence of large numbers of participants and balanced groups ensured that statistical analyses were powerful and accurate. Our study is the first study in the literature to determine parents’ attitudes about vaccination by using the PACV scale and the PARI scale together. The present study was conducted with only mothers as participants; fathers were not included. It has been observed in the literature that a significant proportion of studies investigating VH and VR include a high number of women [30, 54]. Since our aim was to evaluate vaccine attitudes along with parental attitudes, it was thought that it would be more useful to look at it from a single parent’s perspective. More comprehensive studies that include fathers and other caregivers may yield different and broader results. Future studies could benefit from exploring the perspectives of fathers and other caregivers as well, to gain a more comprehensive understanding of VH within families.

Since our study was conducted in a single center where patients came from surrounding provinces, the fact that the participants had similar traditions and culture may have affected the survey results slightly. People who live in the same neighborhood and whose children have similar diseases may meet and influence each other’s thoughts about vaccination. Similar studies to be conducted with participants from all regions of our country may provide different results in terms of ethnic and socio-cultural aspects.

## Conclusion

In this large-scale study, which included children with various childhood diseases of different pathophysiology and healthy children, VH rates among parents were assessed using the PACV survey, and potential solutions were explored by analyzing their attitudes. While VH and VR are frequently discussed in parents of children with ASD, our study identified VH in approximately one-third of parents of children with T1DM, a high-risk group for vaccine-preventable infectious diseases requiring additional vaccinations beyond routine childhood immunization. Notably, these parents attributed their children’s illness to the MMR vaccine, a novel and unexpected finding in the literature.

The child’s CC, having experienced vaccine adverse events, having a child/sibling with CC, parents not having COVID-19 vaccination, and using the internet as an information source stand out as conditions that increase the risk of VH. A multifaceted approach, including targeted educational interventions against misinformation, is necessary, especially for parents with children in high-risk groups. All physicians who follow children with chronic diseases who are at risk, especially for VPDs, should be careful about vaccines. Strengthening healthcare provider-parent communication and promoting evidence-based vaccine information through reliable sources may help reduce hesitancy. Future public health strategies should focus on promoting trust, increasing health literacy, and implementing specific interventions that take into account the psychological and social determinants of VH. It has been observed that a high score on the democratic attitude and equality recognition subscale also reduces the risk of VH. Determining the risk of VH by identifying parental attitudes and proposing solutions may be a more accurate and better-yielding approach. Further research with larger cohorts is therefore recommended in order to develop more effective, data-driven policies aimed at reducing VH and improving vaccination rates in vulnerable populations.

## Electronic supplementary material

Below is the link to the electronic supplementary material.

Supplementary Material 1: Sociodemographic data and vaccine follow-up form

## Data Availability

The anonymized dataset is available from the corresponding author (siyalcin@hacettepe.edu.tr) on reasonable request.
