# Supplementary material for: Vaccine hesitancy among parents of children with chronic diseases of different pathophysiology: a cross-sectional study in Sivas, Türkiye
Source: BMC Public Health. 2025 May 7;25:1683. doi: 10.1186/s12889-025-22797-y (PMC12057203; doi:10.1186/s12889-025-22797-y)
Supplement: Supplementary file 1 — Supplementary Material 1: Sociodemographic data and vaccine follow-up form [file 12889_2025_22797_MOESM1_ESM.rtf]

SOCIODEMOGRAPHIC DATA AND VACCINE FOLLOW-UP FORM

Date: 
Child's Birth Date: 								Age:
Residence:  Province [ ]       District [ ]            Village [ ]
Mother's age:
Morher's education level: Primary school [ ]   Secondary school [ ]     High school [ ]              University and above [ ]  
Mother's employment:
Father's age:
Father's education level: Primary school [ ]      Secondary school [ ]       High school [ ]  University and above [ ]  
Father's employment:  
Household income: Minimum wage and below [ ]  2X the minimum wage   [ ] 3X the minimum wage and more [ ]   
Family type:   [ ] Nuclear        [ ] Extended             [ ] Divorced
In extended families, other members of the household : [ ] Grandparents    [ ]Uncle/aunt        [ ]others
Number of children in the household:………………………………………………………………
Immunisation status of the child:  Fully Vaccinated [ ]   Incompletely vaccinated [ ]   Unvaccinated [ ]   
Were there any adverse effects in vaccination of child?  
[ ] No   [ ] Yes (Explain)..................................................................................................................
Vaccination card: Seen [ ]        Mother's statement [ ]   
Health and vaccination status of siblings (chronic disease, medication used continuously, hospitalisation to be specified) 
1:.........................................................................................................................................................
2:.........................................................................................................................................................
3:.........................................................................................................................................................
4:.........................................................................................................................................................
5: ........................................................................................................................................................
Were there any adverse effects during the vaccination of siblings: 
[ ] No   [ ] Yes (Explain)...................................................................................................................
Has the mother received at least 2 doses of COVID-19 vaccine?  [ ] Yes          [ ] No
Has the father received at least 2 doses of COVID-19 vaccine?  [ ] Yes           [ ] No
Has the mother been vaccinated against tetanus in the last 5 years?  [ ] Yes       [ ] No
Has the father been vaccinated against tetanus in the last 5 years?  [ ] Yes        [ ] No
Has the mother been vaccinated against influenza in the last 5 years?  [ ] Yes     [ ] No
Has the father been vaccinated against influenza in the last 5 years?  [ ] Yes      [ ] No

Child's disease:
[ ] Autism spectrum disorder
[ ] Attention deficit hyperactivity disorder
[ ] Congenital heart disease
[ ] Type 1 Diabetes
[ ] Congenital hypothyroidism
[ ] Familial Mediterranean Fever 
[ ] Other..........................................................................................................................................
The year of the diagnosis:...............................................................................................................
Treatment received:.........................................................................................................................

Where do you get information about vaccinations?
[ ] Doctor-nurse-health personnel   
[ ] Books-journals  
[ ] Internet Websites
[ ] Facebook-Instagram-X
[ ] Family elder or opinion leader
[ ] Other...............................................................................................................................................

(The following questions will be answered by vaccine hesitant parents)
What are your concerns about vaccines?
........................................................................................................................................................................................................................................................................................................................
How do you think this concern can be solved?
........................................................................................................................................................................................................................................................................................................................

Thank you for your participation.
